# Hepatitis B Virus (HBV) Genotype Mixtures, Viral Load, and Liver Damage in HBV Patients Co-infected With Human Immunodeficiency Virus

**DOI:** 10.3389/fmicb.2021.640889

**Published:** 2021-03-03

**Authors:** Alexis Jose-Abrego, Sonia Roman, João Renato Rebello Pinho, Vanessa Fusco Duarte de Castro, Arturo Panduro

**Affiliations:** ^1^Department of Molecular Biology in Medicine, Civil Hospital of Guadalajara, “Fray Antonio Alcalde,” Guadalajara, Mexico; ^2^Health Sciences Center, University of Guadalajara, Guadalajara, Mexico; ^3^LIM-07, Department of Gastroenterology, School of Medicine, Institute of Tropical Medicine, University of São Paulo, São Paulo, Brazil; ^4^Hospital Israelita Albert Einstein, São Paulo, Brazil

**Keywords:** liver fibrosis, hepatitis B virus, co-infection, HBV genotype, HBV genotype mixtures, human immunodeficiency virus

## Abstract

Hepatitis B virus (HBV) co-infection is possible in patients who are positive for human immunodeficiency virus (HIV) since both share similar transmission routes. Furthermore, through the continuous risk of exposure, they potentially can be infected by mixtures of distinct HBV genotypes which can result in the presence of two or more genotypes in a single patient. This study aimed to specify the frequency of mixtures of HBV genotypes and their potential clinic importance in HIV-infected Mexican patients. HBV infection was assessed by serological testing and molecular diagnostics. HBV mixtures were detected by multiplex PCR and DNA sequencing. Liver fibrosis was evaluated using transitional elastography, the Aspartate aminotransferase to Platelets Ratio Index score, and Fibrosis-4 score. Among 228 HIV-infected patients, 67 were positive for HBsAg. In 25 HBV/HIV co-infected patients, 44 HBV genotypes were found: H (50.0%, 22/44), G (22.7%, 10/44), D (15.9%, 6/44), A (9.1%, 4/44), and F (2.3%, 1/44). Among these, 44.0% (11/25) were single genotype, 36.0% (9/25) were dual and 20.0% (5/25) were triple genotype. The most frequent dual combination was G/H (44.4%, 4/9), while triple-mixtures were H/G/D (60.0%, 3/5). The increase in the number of genotypes correlated positively with age (Spearman’s Rho = 0.53, *p* = 0.0069) and negatively with platelet levels (Spearman’s Rho = − 0.416, *p* = 0.039). HBV viral load was higher in triply-infected than dually infected (31623.0 IU/mL vs. 1479.0 IU/mL, *p* = 0.029) patients. Triple-mixed infection was associated with significant liver fibrosis (OR = 15.0 95%CI = 1.29 – 174.38, *p* = 0.027). In conclusion, infection with mixtures of HBV genotypes is frequent in HIV patients causing significant hepatic fibrosis related to high viral load, especially in triple genotype mixtures.

## Introduction

One severe clinical issue in patients with human immunodeficiency virus (HIV) is the high frequency of liver diseases, particularly those caused by chronic HBV infection (Sherman et al., 2017). Intravenous drug use and sexual behavior are some of the important risk factors associated with HBV/HIV co-infections. In hospitalizations related to liver complications, HBV/HIV co-infection is associated with poor clinical outcomes (Rajbhandari et al., 2016). It has also been observed that the progression of chronic HBV to cirrhosis or hepatocellular carcinoma is more rapid in HBV/HIV co-infected patients than in those who are HBV mono-infected (Thio et al., 2002). It is probably linked to an immunosuppression state by reducing the frequency of spontaneous recovery and increasing the risk of chronicity (Seetharam et al., 2014). Also, the clinical management in HBV/HIV co-infected patients can be difficult because they present a more insufficient response to interferon-alpha, have a high risk of HBV post-therapy reactivation, or develop HBV resistance mutations (Di Martino et al., 2002; Mendes-Correa et al., 2010; Tengan et al., 2017).

On the other hand, people with HIV who lack protective immunity present many risk factors exposing them to different HBV genotypes, leading to a mixed infection (Thio et al., 2002). Besides, the common factors mentioned above are social determinants, such as unemployment or living in poverty affect infection risk (Jose-Abrego et al., 2017). These can lead individuals engaging in high-risk behaviors for infection, such as prostitution and intravenous drug use, which may favor infection by different HBV genotypes. Together, with the immunological status of the HIV-infected individual, a mixture of HBV genotypes could develop. Mixtures of genotypes are when more than one HBV genotype is detected in the blood resulting in a simultaneous infection or superinfection (i.e., when a second genotype is detected after a single HBV genotype infection) (Cunningham et al., 2015). HBV genotype mixtures have been reported in some groups, such as patients treated with interferon and injection drug users (Hannoun et al., 2002; Chen et al., 2004). Toan et al. (2006) showed the clinical importance of HBV genotypes mixtures in people negative to hepatitis C virus and HIV. Data on the frequency of mixtures of HBV genotypes among HBV/HIV co-infected patients remains understudied.

Approximately 220,000 persons live with HIV in Mexico. Between 24 and 29% have evidence of HBV chronic infection (Torres-Baranda et al., 2006; Jose-Abrego et al., 2017; The Joint United Nations Programme on HIV/AIDS, 2019). Furthermore, previous studies have reported a high prevalence of occult hepatitis B (OBI) in HIV cohorts. Torres-Baranda et al. (2006) reported an OBI prevalence of 18.4% in West Mexico. In central Mexico, the prevalence of OBI varied between 36% and 49% (Alvarez-Munoz et al., 2014; Enriquez-Navarro et al., 2020). Although the endemic genotype H is common (64%) among HBV/HIV co-infected patients, other genotypes such as G (16%), A2(12%), F1b (4%), and D4(4%) have been reported (Jose-Abrego et al., 2017). This finding suggests that new introductions have occurred, requiring molecular epidemiology studies to decipher if mixed genotypes in HBV infections prevail. Furthermore, in our country, people who inject drugs and are MSM have a high risk of HBV/HIV co-infection (Jose-Abrego et al., 2017). These factors suggest that some people with HIV might acquire infections with different HBV genotypes. Therefore, in this study, we aimed to detect HBV genotypes’ mixtures and their impact on viral load and liver damage in Mexican patients with HIV.

## Materials and Methods

### Study Design and Socio-Demographic Data

**FIGURE 1:**
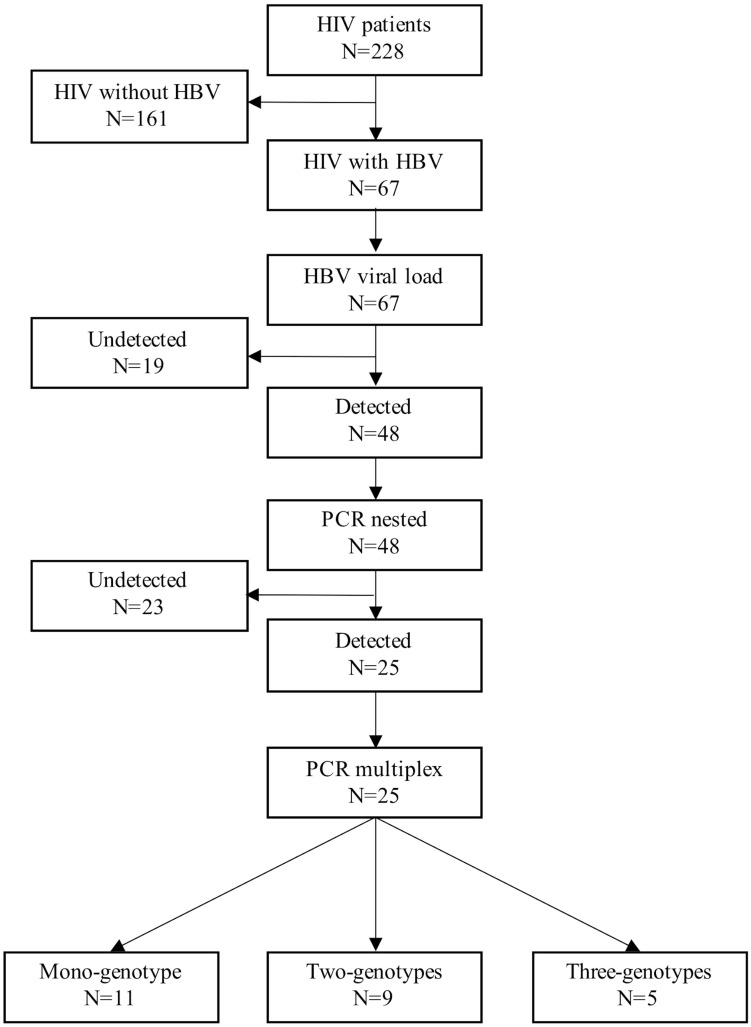
Workflow used to detect mixtures of HBV genotypes in patients with HIV. First, serum samples were collected from all patients. These samples were analyzed by ELISA to evaluate the presence of HBsAg. Next, in the positive samples from the previous step, the viral load levels were measured by real-time PCR. A nested PCR was used to confirm HBV infection in the samples with detectable viral load. After that, a multiplex PCR was used to detect the predominant HBV genotypes in Mexico. Finally, the products were sequenced and genotyped by Sanger’s method and phylogenetic analysis, respectively.

This cross-sectional study included 228 patients with a serological or molecular diagnosis of HIV. All patients were attended at the Department of Molecular Biology in Medicine and the HIV Clinic of the Civil Hospital of Guadalajara “Fray Antonio Alcalde” from January 2014 to December 2016 (Jose-Abrego et al., 2017). Written informed consent was obtained from all participants. Demographic and clinical information, such as gender, age, HIV viral load, and the combination antiretroviral therapy (cART) received during recruitment, were obtained from the patients’ medical records. The levels of liver enzymes, platelets, albumin, CD4 cells, and CD8 cells were obtained from our hospital’s general laboratory. Also, hepatitis C virus (HCV) infection was assessed by the presence of anti-HCV antibodies (AxSYM^®^, Abbott Laboratories, Abbott Park, IL, United States). The methodological summary of this study is shown in Figure 1.

### HBV Serological Markers and Viral Load

All serum samples were tested for hepatitis B surface antigen (HBsAg) using an enzyme-linked immunosorbent assay (MONOLISA^TM^ HBsAg Ultra, Bio-Rad, Poincaré, MC, France) according to the manufacturer’s instructions. In samples positive to HBsAg, HBV viral load was quantified by real-time polymerase chain reaction (RT PCR) (COBAS^®^ Ampliprep/COBAS^®^ TaqMan^®^ HBV test (Roche, V2.0), which has a detection limit between 20 and 170 million IU/mL (1IU corresponding to 5.82 genomic copies). Levels ≥ 2,000 IU/mL was considered as high viral load and <2,000 IU/mL as low viral load.

### Extraction of HBV DNA

In the samples with detectable HBV viral load, total viral DNA was extracted using the QIAamp DNA Mini Kit (Qiagen Science, Hilden, Germany) according to the manufacturer’s instructions. Additionally, a carrier DNA (1 μg/μL) (Qiagen Science, Hilden, Germany) and an elution volume of 50 μL were used to improve the extraction yield.

### Qualitative Detection of HBV DNA

The yield of DNA extracts was tested by nested PCR. The amplification reaction was prepared with 5 μL of DNA, 1 μL of each primer (10 μM) (Supplementary Table 1), 0.5 μL of dNTPs mix (10 mM each), 1 μL of MgCl_2_ (50 mM), 2.5 μL of PCR buffer (10×), 13.8 μL of water and 0.2 μL of Taq DNA polymerase (5U/μL) (Invitrogen, San Diego, CA, United States) in a final volume of 25 μL. The first round amplified a 418-bp fragment of the surface gene under the following conditions: 94°C for 3 min followed by 40 cycles of 94°C for 30 s, 55°C for 30 s, 72°C for 30 s, with a final extension step of 72°C for 5 min. The second round amplified a 232-bp product under the same conditions.

### Identification of HBV Genotype Mixtures

DNA extracts were genotyped by multiplex PCR to identify the most predominant HBV genotypes in Mexico (H, G, A, and D) (Roman et al., 2014). These PCRs amplified different parts of the polymerase gene, a 370 bp fragment for genotype A, 147 bp for genotype D, and 279 bp for H, while a 584 bp fragment of the core gene was amplified for genotype G (Supplementary Table 1). The reaction was prepared with 2 μL of DNA extract, 1 μL of each genotype-specific primer set (10 μM), 8.5 μL of water, and 12.5 of 2× Hot StarTaq Master Mix (Qiagen Science, GmbH, Hilden, Germany). Thermal cycling conditions were as followed: preheating at 95°C, 15 min and 35 amplification cycles at 94°C for 1 min, 60°C for 1 min, 72°C for 2 min, and a final extension at 72°C for 10 m. DNA amplicons were run on a 2% TBE-agarose gel stained with SYBR Safe DNA gel (Invitrogen, Carlsbad, CA, United States) and evaluated under UV light. The sizes of PCR products were estimated based on the migration pattern of a 50-bp DNA ladder (Invitrogen, Carlsbad, CA, United States). Quality control procedures included adding positive and negative controls in each PCR assay, and standard precautions were taken to avoid cross-contamination. The primer sequences designed for genotypes H and G were previously published (Panduro et al., 2013**;** Roman et al., 2014), whereas primers for genotypes A and D were used as published by Kirschberg et al. (2004) (Supplementary Table 1). Mixtures of HBV genotypes were defined as finding two or more products in agarose gel, and the genotype was determined according to the band’s size of the amplified DNA.

### HBV-DNA Sequencing and Phylogenetic Analysis

Hepatitis B virus genotypes identified by multiplex PCR were confirmed by DNA Sanger sequencing. The bands were cut and purified with Illustra GFX PCR DNA and Gel Band Purification Kit (GE Healthcare, Pittsburgh, PA, United States). The bidirectional sequencing reaction was prepared with 1 μL of each purified band, 2 μL of each genotype-specific primer (1 μM), 1 μL of sequencing buffer (5×), 4 μL of water, and 2 μL of BigDye^®^ Terminator v3.1 (Applied Biosystems, Foster City, CA, United States). Thermal cycling conditions were as followed: preheating at 96°C, 1 min and 35 amplification cycles at 96°C for 10 s, 50°C for 5 s, 60°C for 4 min, and a final extension at 60°C for 1 m. Finally, the products were analyzed on an ABI 3130 DNA Sequencer (Applied Biosystems, Foster City, CA, United States). HBV DNA sequences were genotyped by phylogenetic analysis using the Molecular Evolutionary Genetic Analysis software, MEGA 10.0^1^. The reference sequences used in this study were: X02763, X51970, and AF090842 for genotype A; AF100309 and AB033554 for genotype B; AB014381, X04615, and AY123041 for genotype C; M32138, X65259, and X85254 for genotype D; X75657 and AB032431 for genotype E; X69798, AF223965, and AB036910 for genotype F; AB064310 and AB625342 for genotype G; and AY090460, AB516395, and AB516394 for genotype H. ClustalW method was used for multiple sequence alignment, while Maximum Likelihood method was used to construct the phylogenetic tree with a bootstrap test of 1000 replicates. The substitution model was General Time Reversible with Gamma Distribution and Invariants Sites (GTR + G + I). Accession numbers submitted to GenBank^2^ of the HBV samples identified in this study appear as MH780886-MH780888, MF150683 for genotype A2; MH780889-MH780897, MF150677 for genotype G; MH780898-MH780909, MF150649-MF150650, MF150658-MF150659, MF150666-MF150669, MF150672-MF150673 for genotype H; MF150685 for genotype D4 and MF150688 for genotype F1b. An alternative sequencing strategy was required for the F1b and D4 sub-genotypes (Mendes-Correa et al., 2010). This strategy was necessary as the D multiplex products were untypeable by direct sequencing. While, for the F1b sub-genotype, we did not expect to find genotype F in Mexico.

### Assessment of Liver Damage

Liver fibrosis was assessed through three non-invasive methods: transitional elastography (TE), Aspartate aminotransferase (AST) to Platelets Ratio Index score (APRI), and Fibrosis-4 score (FIB-4). TE was carried out using a FibroScan^®^ instrument (Echosens, Paris, France). Liver stiffness measurement (LSM) expressed in kiloPascals (kPa) was performed by the same qualified physician. LSM was considered reliable if ten successful measurements were obtained, and the success rate was >80%. APRI was calculated considering the upper limit of normal (ULN) serum AST for men equal to 40 IU/in the equation AST *IU/L*/(ULN AST/(Platelets count 10^9^*L*) ×100 (Sandrin et al., 2003; Borsoi Viana et al., 2009). FIB-4 was calculated with the equation (Age-*years*) (AST level *IU/L*)/(Platelets count 10^9^*L*) (ALT)^1/2^ *IU/L*) (Kim et al., 2010). APRI cut-off value ≥ 0.7 was considered significant hepatic fibrosis, while FIB-4 cut-off value ≥ 3.25 was interpreted as advanced fibrosis (Sterling et al., 2006; Lin et al., 2011). Also, LSM was classified as F1 (<7.1 kPa), F2 (≥7.1 kPa), F3 (≥9.3 kPa), and F4 or cirrhosis (≥12.3 kPa). F1-F2 was taken as mild liver fibrosis and F3-F4 as advanced liver fibrosis (Hawkins et al., 2013). Levels of liver enzymes, platelets, HIV viral load, CD4, and CD8 lymphocytes were attained by routine analysis from the hospital’s Central Laboratory.

### Statistical Analysis

Continuous variables were expressed as the median and interquartile range, while categorical variables were expressed in frequency or percentage. Categorical variables were compared using the *χ*2 test or Fisher’s exact test where appropriate. Data normality was calculated using the Shapiro–Wilk test. Non-parametric continuous variables were tested by Mann–Whitney or Kruskal–Wallis test. All correlations were tested with Spearman’s Rho, and the cut-off of quantitative values was calculated with Receiver Operating Characteristic (ROC) curve analysis. All statistical tests were performed with the Statistical Program for Social Sciences software (SPSS 22.0, IBM, Inc., Armonk, NY, United States). For all statistical tests, a *P*-value of <0.05 was considered significant.

### Ethics

The Ethics Committee of the University Center for Health Sciences of the University of Guadalajara approved this protocol (#CI-07218). Furthermore, this study was carried out by the principles of the Helsinki Declaration.

## Results

### General Characteristics of the Study Population

A total of 228 patients with HIV were analyzed; most of them were men (86.4%, 197/228) between 32 and 46 years of age (Table 1). At the time of this study, 82.0% (187/228) of the participants were on antiretroviral therapy, with a median HIV viral load of 40.0 IU/mL [Interquartile range (IQR): 39.0–326.0 IU/mL]. Among the 187 treated patients, the medical records revealed that cART was tailored individually in which none were monotherapy. All received at least one drug that is known to affect the HBV’s life cycle, such as tenofovir, lamivudine, or emtricitabine. However, the most frequent combinations were tenofovir + emtricitabine + efavirenz in 39.6% (74/187), followed by tenofovir + emtricitabine + lopinavir + ritonavir in 11.2% (21/187) and 49.2% (92/187) received diverse unique combinations with these and other antiviral drugs. Also, 62 individuals out of 132 had evidence of hepatitis C virus (HCV) infection (Table 2). Overall, liver injury was detected in 24.6% (31/126), of which 18.3% (24/131) had significant liver fibrosis, 6.9% (9/130) had advanced fibrosis, and with liver stiffness, 22.5% (11/71) had advanced liver fibrosis (F3-F4) (Table 3).

**TABLE 1:** Demographic data of patients with HIV and HIV/HBV.

	**Total (*N* = 228)**	**HIV alone (*N* = 161)**	**HIV/HBV (*N* = 67)**	***p*-value**
**Gender**				
Female	31 (13.6%)	30 (18.6%)	1 (1.5%)	<0.001^1^
Male	197 (86.4%)	131 (81.4%)	66 (98.5%)	
**Age (years)**				
Mean (SD)	39.9 (10.9)	39.7 (11.7)	39.2 (9.7)	0.758^2^
**Age group (years)**				
<15	1 (0.4%)	1 (0.6%)	0 (0.0%)	1.000^3^
15–20	4 (1.8%)	4 (2.5%)	0 (0.0%)	0.322^3^
21–25	10 (4.5%)	7 (4.4%)	3 (4.5%)	1.000^3^
26–30	32 (14.3%)	23 (14.6%)	9 (13.6%)	1.000^3^
31–35	41 (18.3%)	29 (18.4%)	12 (18.2%)	1.000^3^
36–40	47 (21.0%)	29 (18.4%)	18 (27.3%)	0.151^3^
41–45	29 (12.9%)	21 (13.3%)	8 (12.1%)	1.000^3^
46–50	24 (10.7%)	17 (10.8%)	7 (10.6%)	1.000^3^
51–55	17 (7.6%)	11 (7.0%)	6 (9.1%)	0.789^3^
56–60	9 (4.0%)	8 (5.1%)	1 (1.5%)	0.288^3^
61–65	5 (2.2%)	4 (2.5%)	1 (1.5%)	1.000^3^
>66	5 (2.2%)	4 (2.5%)	1 (1.5%)	1.000^3^

*^1^Pearson’s Chi-squared test.*

*^2^*T*-student test.*

*^3^Fisher Exact test.*

*Age had a normal distribution. SD, standard deviation; HBV, hepatitis B virus; HIV, human immunodeficiency virus.*

**TABLE 2:** Baseline clinical characteristics of patients with HIV and HIV/HBV.

	**Total (*N* = 228)**	**HIV alone (*N* = 161)**	**HIV/HBV (*N* = 67)**	***p*-value**
**cART**				
Without cART	32 (14.0%)	25 (15.5%)	7 (10.4%)	0.371^1^
With cART	196 (86.0%)	136 (84.5%)	60 (89.6%)	
**HBV viral load (IU/mL)**				
Median (Q1, Q3)	145.0 (20.0, 18312.5)	NA	145.0 (20.0, 18312.5)	
**HIV viral load (IU/mL**)				
Median (Q1, Q3)	40.0 (39.5, 303.5)	40.0 (40.0, 388.0)	40.0 (34.8, 137.5)	0.520^2^
**HCV infection**				
Negative	132 (68.0%)	73 (55.7%)	59 (93.7%)	<0.001^1^
Positive	62 (32.0%)	58 (44.3%)	4 (6.3%)	
**ALT IU/L**				
Median (Q1, Q3)	27.0 (20.0, 44.0)	24.5 (17.0, 36.2)	30.0 (22.0, 52.5)	0.006^2^
**AST IU/L**				
Median (Q1, Q3)	30.0 (24.0, 43.0)	30.5 (24.0, 48.5)	29.0 (24.0, 40.0)	0.734^2^
**GGT IU/L**				
Median (Q1, Q3)	41.0 (24.2, 61.8)	44.0 (29.0, 82.0)	34.0 (24.0, 48.0)	0.126^2^
**ALP IU/L**				
Median (Q1, Q3)	97.0 (80.2, 126.0)	95.0 (73.0, 135.0)	100.0 (85.0, 119.5)	0.996^2^
**Platelets cells/μL**				
Median (Q1, Q3)	226.0 (181.0, 293.0)	232.0 (179.0, 309.0)	224.5 (187.5, 281.0)	0.697^2^
**Albumin g/dL**				
Median (Q1, Q3)	3.8 (3.2, 4.1)	3.6 (2.6, 3.9)	3.9 (3.7, 4.1)	<0.001^2^
**CD4 cells/mm3**				
Median (Q1, Q3)	299.0 (179.2, 547.8)	273.0 (92.2, 447.2)	339.0 (236.5, 607.5)	0.007^2^
**CD8 cells/mm3**				
Mean (SD)	954.7 (553.4)	908.3 (555.2)	1039.0 (565.2)	0.129^3^

*^1^Pearson’s Chi-squared test.*

*^2^Wilcoxon-Mann-Whitney test.*

*^3^*T*-student test.*

*Patients with HCV were excluded for the comparison of laboratory data. CD8 levels had a normal distribution. NA, not available; SD, standard deviation; cART, combination antiretroviral therapy; HBV, hepatitis B virus; HCV, hepatitis C virus; HIV, human immunodeficiency virus. ALT, alanine aminotransferase; AST, aspartate aminotransferase; GGT, gamma-glutamyl transferase; ALP, alkaline phosphatase; CD4, lymphocyte CD4 count; CD8, lymphocyte CD8 count; Q1, quartile 1; Q3, quartile 3.*

**TABLE 3:** Baseline non-invasive markers of liver damage in HIV and HBV/HIV patients.

	**Total (*N* = 228)**	**HIV alone (*N* = 161)**	**HIV/HBV (*N* = 67)**	***p*-value**
**APRI value**				
Median (Q1, Q3)	0.3 (0.2, 0.5)	0.3 (0.2, 0.6)	0.3 (0.2, 0.5)	0.921^2^
**FIB-4 value**				
Median (Q1, Q3)	0.9 (0.7, 1.4)	1.1 (0.7, 1.6)	0.9 (0.7, 1.3)	0.407^2^
**Liver stiffness (Kpa)**				
Median (Q1, Q3)	6.1 (4.8, 8.6)	6.1 (4.5, 9.5)	6.8 (5.0, 8.2)	0.766^2^
**Liver inflammation**				
ALT or AST or GGT < 40 IUı/mL	95 (75.4%)	50 (70.4%)	45 (81.8%)	0.141^1^
ALT or AST or GGT ≥ 40 IUı/mL	31 (24.6%)	21 (29.6%)	10 (18.2%)	
**APRI class**				
Without significant liver fibrosis (<0.7)	107 (81.7%)	57 (79.2%)	50 (84.7%)	0.411^1^
With significant liver fibrosis (≥0.7)	24 (18.3%)	15 (20.8%)	9 (15.3%)	
**FIB-4 class**				
Without advanced fibrosis (<3.25)	121 (93.1%)	66 (91.7%)	55 (94.8%)	0.480^1^
With advanced fibrosis (≥3.25)	9 (6.9%)	6 (8.3%)	3 (5.2%)	
**Liver stiffness class**				
F1	44 (62.0%)	16 (66.7%)	28 (59.6%)	0.614^3^
F2	11 (15.5%)	1 (4.2%)	10 (21.3%)	0.262^3^
F3	5 (7.0%)	4 (16.7%)	1 (2.1%)	0.042^3^
F4	11 (15.5%)	3 (12.5%)	8 (17.0%)	0.739^3^

*^1^Pearson’s Chi-squared test.*

*^2^Wilcoxon-Mann-Whitney test.*

*^3^Fisher Exact test.*

*Patients with HCV were excluded from the analyses. HBV, hepatitis B virus; HIV, human immunodeficiency virus; ALT, alanine aminotransferase; AST, aspartate aminotransferase; GGT, gamma-glutamyl transferase; APRI, AST to Platelet Ratio Index; FIB-4, fibrosis-4 score; Q1, quartile; Q3, quartile 3.*

### HBV Infection Status

Of the 228 HIV individuals, 67 were positive for HBsAg; these were considered HBV/HIV co-infected patients (Table 2). HIV/HBV co-infection cases were detected between 21 and 25 years, while the maximum age was reached in persons between 36 and 40 years. Overall, the HBV viral load was detected in 71.6% (48/67) of the cases, with a median HBV viral load of 145.0 IU/mL (IQR: 20.0–166616.0 IU/mL). A low HBV viral load (<2,000 IU/mL) was detected in 66.6% (32/48) of HBV/HIV patients, while 33.3% (16/48) had a high HBV viral load (≥2,000 IU/mL). In this study, 89.1% (57/67) of these HBsAg positive cases were under antiretroviral treatment. Among these, 43.9% (25/57) were treated with tenofovir + emtricitabine + efavirenz, followed by 10.5% (6/57) receiving tenofovir + emtricitabine + lopinavir + ritonavir, and 45.6% (26/57) received as mentioned above several unique combinations with other drugs. The liver enzyme profile was similar between HIV alone (without HBV or HCV) and HBV/HIV patients. However, ALT levels were higher in HBV/HIV co-infection than HIV alone (30 IU/L (IQR: 22 – 52.5 IU/L) vs. 24.5 (IQR: 17.0–36.2), *p* = 0.006). Among the HBV/HIV cases, the frequency of liver inflammation was 18.2% (10/55), 15.3% (9/59) had significant liver fibrosis, 5.2% (3/58) had advanced fibrosis, and with LSM, 19.1% (9/47) had advanced liver fibrosis (F3-F4) (Table 3).

### Distribution of HBV Genotypes and Mixtures

**FIGURE 2:**
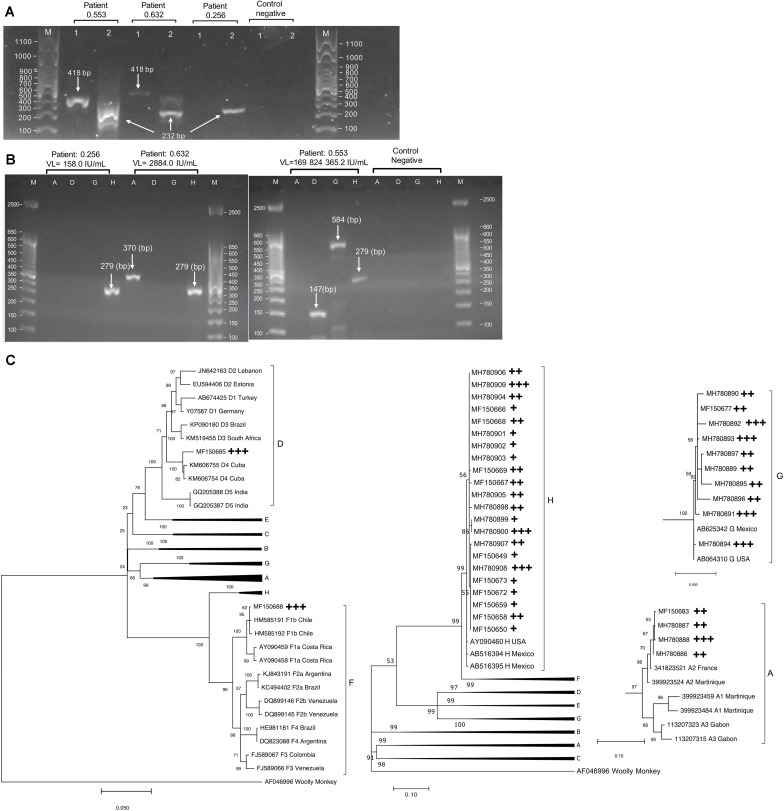
**(A)** Different size bands were expected for the nested PCR. In patients with high viral loads, both bands’ presence is evident (418 bp and 232 bp), but only the second band is visualized in patients with relatively low viral load. **(B)** Multiplex PCR results, after the molecular weight marker, the first four lanes show a patient’s results with one HBV genotype (patient 0.256), while the next four lines show a mixture of two HBV genotypes (patient 0.632). Finally, the lanes of patient 0.553 show a typical result of a person infected with three HBV genotypes. Based on the band intensity, we noted that each HBV genotype has a different viremia level, and one genotype is usually dominant over others. **(C)** Phylogenetic trees are used for genotyping and sub-genotyping of sequenced multiplex PCR products. The numbers in the trees represent the bootstrap values of each node. Since different genomic locations classified each genotype, four phylogenetic trees were built. The cross symbol indicates the type of infection in which the genotype was detected: +, single-infected sample; ++, dual-infection sample; +++, triple-infection sample.

Among the 48 patients with detectable HBV viral load, 25 cases were positive to the nested PCR assay in which 44 HBV genotypes were detected by multiplex PCR (Figures 2A, B). According to our methodology, the minimum viral load for HBV genotyping was 82.0 IU/mL [Area Under the Curve, (AUC) = 0.93, *p* = 5 × 10^–5^] (Supplementary Figure 1A). The predominant HBV genotype was H (50.0%, 22/44), followed by G (22.7%, 10/44), D (15.9%, 6/44), A (9.1%, 4/44), and F (2.3%, 1/44). Figure 2C illustrates the phylogenetic tree built for each genotype of the 38 strains confirmed by direct DNA sequencing. All genotypes A were sub-genotyped as A2, one genotype F was classified as F1b, and one D was D4. Among typeable samples, 44.0% (11/25) had a single genotype (10 were H and one G), whereas 36.0% (9/25) had dual-mixtures (four were G/H), two were D/H, two were A2/H, and one was A2/G) Moreover, 20.0% (5/25) were triple-mixtures (three were H/G/D, one was A2/D/G, and one was H/D/F1b). G/H (44.4%, 4/9) and H/G/D (60.0%, 3/5) were the most frequent genotype mixtures. Nineteen out of 25 genotyped cases were under cART in which five mono-infected, three dual-mixed, and one triple-mixed were treated with tenofovir + emtricitabine + efavirenz. Two triple-mixed cases were treated with tenofovir + emtricitabine + lopinavir + ritonavir whereas three mono-infected, four dually infected and one triple-mixed case received diverse combinations. Interestingly, ten patients had high HBV viral loads (≥2,000 IU/mL) despite receiving a tenofovir-based regimen; five were mono-infected, (four H and one G), one dual-mixed (A/G), and four were triple-mixed (two D/G/H, one A/D/G, and one D/H/F1b).

### Relationship Between HBV Mixtures and Clinical Characteristics

**FIGURE 3:**
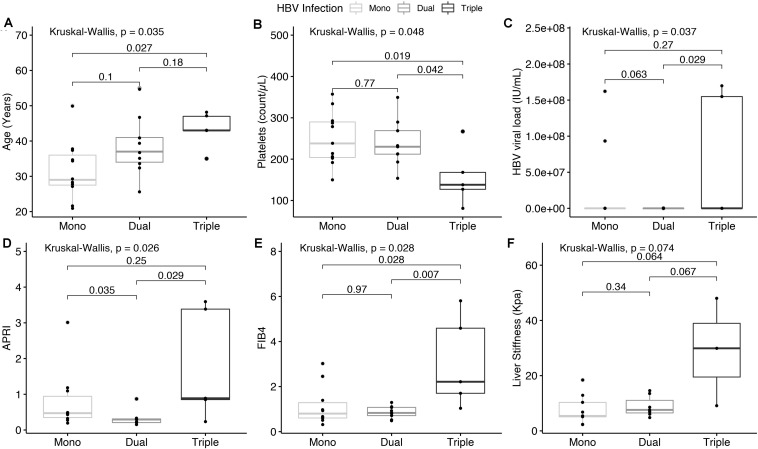
**(A)** Shows a significant relationship between increasing age and the presence of a mixture of HBV genotypes. **(B)**, Platelet levels decreased while increasing the number of HBV genotypes. **(C)** Shows viral load levels by type of mixes. **(D–F)** Shows the comparison of the degree of fibrosis in patients with one, two, and three HBV genotypes, evaluated through the APRI, FIB-4, and LSM, respectively. The boxes’ middle lines represent the median, while the outer lines represent 25 and 75 quartiles. Black dots indicate each patient studied. LSM, liver stiffness measurement.

We compared different clinical parameters as dependent variables relative to the number of HBV genotypes (Figures 3A–C and Supplementary Table 2), finding a positive correlation between increasing age and mixtures (Spearman’s Rho = 0.53, *p* = 0.0069) (Figure 3A). Interestingly, the median age of patients with two HBV genotypes was 37.0 years, while the age-associated with triple-mixed cases was ≥42.0 years (AUC = 0.805, *p* = 0.038) (Supplementary Figure 1B). Moreover, platelet levels were negatively correlated with the increase in the number of HBV genotypes (Spearman’s Rho = − 0.416, *p* = 0.039) (Figure 3B). HBV viral load (31623.0 IU/mL vs. 1479.0 IU/mL, *p* = 0.029) was higher in triple-mixtures than dual-mixtures (Figure 3C). Regarding liver damage, the degree of fibrosis was similar between single and dual genotype mixtures (LSM, *p* = 0.34, FIB-4, *p* = 0.97), while the liver fibrosis grade was significantly higher in triple-mixtures than dual-mixtures (APRI, *p* = 0.029; FIB-4, *p* = 0.007) (Figures 3D–F). Based on APRI, patients with HIV and three HBV genotypes had a high risk of presenting significant liver fibrosis (OR = 15.0 95%CI = 1.29 – 174.38, *p* = 0.027) compared with dual-mixtures or single genotype infection (Table 4).

**TABLE 4:** Association between the number of HBV genotypes and liver fibrosis in patients with HIV/HBV co-infection.

	**APRI**			**FIB-4**			**LSM**		
	**Without significant liver fibrosis (<0.7)**	**With significant liver fibrosis (≥0.7)**	**Odds ratio (95% CI) *p*-value^1^**	**Without advanced fibrosis (<3.25)**	**With advanced fibrosis (≥3.25)**	**Odds ratio (95% CI) *p*-value^1^**	**Mild liver fibrosis (F1-F2)**	**Advanced liver fibrosis (F3-F4)**	**Odds ratio (95% CI) *p*-value^1^**
**HBV genotypes**									
Single *n* (%)	7 (43.8)	3 (37.5)	0.771 (0.136–4.391) *p* = 0.561	10 (45.5)	0 (0.0)	0.857 (0.692–1.062) *p* = 0.330	6 (50.0)	3 (42.9)	0.750 (0.115–4.898) *p* = 0.570)
Dual *n* (%)	8 (50.0)	1 (12.5)	0.143 (0.014–1.444) *p* = 0.087	9 (40.9)	0 (0.0)	0.867 (0.711–1.057) *p* = 0.380)	5 (41.7)	2 (28.6)	0.560 (0.076–4.144) *p* = 0.474
Triple *n* (%)	1 (6.3)	4 (50)	15.0 (1.290–174.386) *p* = 0.027	3 (13.6)	2 (100.0)	1.667 (0.815–3.409) *p* = 0.036)	1 (8.3)	2 (28.6)	4.4 (0.319–60.614) *p* = 0.296)

*^1^Fisher Exact test.*

*APRI, AST to Platelet Ratio Index; FIB-4, fibrosis-4 score; LSM, liver stiffness measurement.*

## Discussion

Previously, we reported the frequency of HBV infection and its risk factors associated with co-infection in this HIV cohort (Jose-Abrego et al., 2017). However, the frequency of liver fibrosis and the proportion of patients with HBV genotype mixtures remained unknown. In this study, the assessment of LSM with transitional elastography detected more cases of liver fibrosis than APRI or FIB-4. Overall, the frequency of advanced liver fibrosis in Mexican patients with HIV was 22.5%, while among HBV/HIV co-infected patients were 19.1%. This proportion was slightly lower than that found in two HBV/HIV co-infected Nigerian studies with frequencies of 22.1 and 22.6% each. Using liver biopsy, Sterling and colleges reported 22% of advanced fibrosis in populations from the United States and Canada (Hawkins et al., 2013; Grant et al., 2017; Sterling et al., 2019). The high frequency of advanced liver fibrosis may be due to inadequate evaluation and diagnostics of hepatitis B in our population. Furthermore, the high frequency of asymptomatic and OBI (Torres-Baranda et al., 2006; Enriquez-Navarro et al., 2020) may facilitate the non-detection of co-infections with other hepatotropic viruses. Alcohol consumption, drug abuse, and potential hepatotoxicity of cART drugs can also be involved (Neff et al., 2006). In regards to liver enzymes, significantly higher levels of ALT but not AST or GGT were found in the HIV group compared to the HIV/HBV co-infected patients. This finding may be due to an insufficient sample size per group to achieve statistical differences. Another reason from a clinical viewpoint, it is known that altered ALT levels or transaminitis is a marker of liver damage than AST whereas abnormal GGT is seen in cases of alcoholic liver disease. In the HIV mono-infected group, high ALT levels could be due to several factors such as medication-related hepatotoxicity, alcohol abuse, drug use, non-alcoholic steatohepatitis or HIV itself.

Our results provide evidence for reconstructing a part of HBV infection’s natural history in patients with HIV. It is known that HIV patients are infected near the age of 15 (Wilson et al., 2010). Later, due to injection drug use or high-risk sexual behavior including men who have sex with men (MSM) (Jose-Abrego et al., 2017), they are infected with hepatitis B between 21 and 25 years. In our cohort, no patient was positive to HBsAg before the age of 20, the first cases of HBV/HIV co-infection (4.5%) occurred around 21, then the maximum frequency of HBV/HIV co-infection was reached at 36–40 years (27.3%). These data suggest that HIV infection occurs before HBV infection. Future longitudinal studies are necessary to demonstrate or refute this observation. Nonetheless, if patients co-infected with HBV/HIV do not change their risk factors, they could acquire HBV genotypes mixtures. Furthermore, our findings show that HIV patients could be co-infected with two HBV genotypes near the age of 37 and up to three HBV genotypes at older ages (42 years). Using PCR multiplex and sequencing, we found that 56% of HIV patients with typeable HBV were infected with more than one HBV genotype. This frequency is higher than those reported in Swiss people and the Netherlands, with 14 and 37%, respectively (Van Der Kuyl et al., 2013**;** Bihl et al., 2015). This difference is mainly due to our study population’s characteristics, who are people with very low-income. Many of them are sex workers, and most do not have access to social security (Jose-Abrego et al., 2017). Another difference with those studies is the type of mixture. A/G mixtures predominate in European HBV/HIV co-infected people, whereas H/G mixtures were predominant in our results. The reason is that genotype H is endemic to Mexico, and genotype G is strongly related to the MSM population as well (Panduro et al., 2013; Roman and Panduro, 2013). Also, we identified five HIV patients who were infected with three HBV genotypes. This evidence indicates that HBV genotype mixtures may be more common than expected in populations with HIV from Mexico and Europe.

In patients without HIV, the mixture of HBV genotypes has been associated with different clinical outcomes, especially high viral load levels (Toan et al., 2006). In this HBV/HIV cohort, high viral load levels predominate in patients with three genotypes than those with dual or single genotype infection. We did not find a clear association with viral load in patients with two genotypes, probably due to tenofovir’s or lamivudine’s antiviral effect. The molecular mechanism explaining the high HBV viral load in patients with mixtures has been documented in *in vitro* models showing that different genotypes interact to increase their replication. For example, genotype G uses the core promoter and core protein from sub-genotype A2 to replicate efficiently. Also, genotype G is abundantly expressed when co-transfected with a construct that expresses the genotype D core protein. D. This occurs because the core promoter of genotype G cannot generate enough core protein for its replication and it may also be defective, affecting the synthesis of virions (Sakamoto et al., 2013). It is plausible that genotype H uses the same mechanism since the replication of genotype G in chimeric mice is enhanced when co-infected with genotype H (Tanaka et al., 2008). Interestingly, our study found patients with G/H, G/A2, and H/G/D mixtures suggesting that in some HIV patients, the high HBV viral load may be due to HBV genotypes mixtures.

This study found a negative correlation between platelet levels and the increase of HBV genotypes; this is understandable as thrombocytopenia is one of the most frequent complications in patients with advanced liver disease (Nusrat et al., 2014). Also, we associated the presence of three HBV genotypes with significant liver fibrosis. The liver damage linked to HBV mixtures could be explained from an immunological viewpoint. First, the increase of viral load due to interaction among HBV genotypes can activate resident liver macrophages. Then, these cells release cytokines, which recruit more macrophages and induce hepatic stellate cell activation. Finally, these signs stimulate the extracellular matrix’s proliferation and deposition, triggering fibrosis development (Koyama and Brenner, 2017). If the immune response or antiviral therapy does not control the infection, people with a mixture of HBV genotypes could eventually develop cirrhosis or liver cancer. In this context, our results indicate that HIV patients may be living with two or more genotypes that could have been acquired simultaneously or through successive contacts. This data is relevant because HBV mono-infected individuals (without HIV), such as heterosexual individuals with multiple partners, people born in HBV endemic areas, or patients under hemodialysis (Trepo et al., 2014), could also present a mixture of HBV genotypes. Perhaps, these groups may be at risk of developing liver damage related to mixtures, as in our HIV cohort. This highlights the importance of assessing the presence of a mixture of HBV genotypes in other risk groups.

Another clinical importance of HBV genotype mixtures is that it can impact treatment outcomes. During treatment, lamivudine, emtricitabine, or tenofovir may only suppress sensitive HBV genotypes, favoring viral persistence due to antiviral resistance mutations (Mendes-Correa et al., 2010; Singh et al., 2017). In this study, four triply-infected patients and one dually infected case had high HBV viral load (≥2,000 IU/mL) despite receiving tenofovir combined with lamivudine or emtricitabine. This finding suggests that in some HBV/HIV co-infected patients, virological failure may be caused by the presence of mixtures of HBV genotypes. However, in this study, the frequency of potential resistance mutations was not evaluated. Thus, further research will be necessary to increase the number of mixed-genotype cases and evaluate the presence of antiviral resistance mutations.

In addition to the risk of fibrosis, high viral loads, and the emergence of resistance mutations, HBV genotype mixtures may play a key role in the origin of new viral strains. This event can occur when a cell is infected with different HBV genotypes that exchange genetic information, resulting in a recombinant genotype (Simmonds and Midgley, 2005). Whole-genome studies have reported HBV recombinant genotype infection in China (Liu et al., 2017), South Africa (Matlou et al., 2019), Japan (Kojima et al., 2015), Argentina, and Brazil (Araujo et al., 2013), particularly in patients with HIV. Currently, a total of 24 phylogenetic recombinant forms are recognized (Simmonds and Midgley, 2005). If recombination confers replicative and spreading advantages, this new virus could fixate in a population and follow its evolutionary path independent of its precursor genotypes, giving rise to new HBV genotypes.

On the other hand, HBV genotypes found in mixtures could accelerate the appearance of mutations (Lok et al., 2000). This event could be due to antivirals that rapidly change the intracellular environment and inhibit viral replication. In response to this selective pressure, HBV genotypes accumulate mutations in the reverse transcriptase domain of the polymerase gene that allow them to change their protein structure and escape antivirals (Locarnini and Yuen, 2010). These features suggest that HBV evolution can occur very quickly when natural selection is robust. Under this approach, the presence of mixtures of HBV genotypes would have biological importance, functioning as a mechanism of HBV diversity.

## Conclusion

In conclusion, a high frequency of advanced liver fibrosis was detected in Mexican patients with HIV. Among the HBV/HIV co-infected, we confirmed the presence of a mixture of HBV genotypes. This infection type was associated with significant liver fibrosis and high viral load levels, especially when three HBV genotypes were detected.

## Data Availability Statement

The datasets presented in this study can be found in online repositories. The names of the repository/repositories and accession number(s) can be found below: https://www.ncbi.nlm.nih.gov/genbank/, MH780886-MH780897 https://www.ncbi. nlm.nih.gov/genbank/, MF150683 https://www.ncbi.nlm.nih.gov/genbank/, MF150677 https://www.ncbi.nlm.nih.gov/genbank/, MH780898-MH780909 https://www.ncbi.nlm.nih. gov/genbank, MF150649-MF150650 https://www.ncbi.nlm.nih.gov/genbank/, MF150658-MF150659 https://www.ncbi.nlm.nih.gov/genbank/, MF150666-MF150669 https://www.ncbi.nlm. nih.gov/genbank/, MF150672-MF150673 https://www.ncbi.nlm.nih.gov/genbank/, MF150685-MF150688.

## Ethics Statement

The studies involving human participants were reviewed and approved by the Ethics Commitee of the University Center for Health Sciences of the University of Guadalajara (#CI-07218). Written informed consent to participate in this study was provided by the participants or the participants’ legal guardian/next of kin.

## Author Contributions

AP and SR: conceptualization. AP, SR, JR, and VFDC: methodology. VFDC: validation. AJ-A, SR, and AP: formal analysis and investigation. AJ-A: data curation and writing-original draft preparation. SR, AP, and JR: writing-review and editing. SR: supervision. AP: funding acquisition. All authors contributed to the article and approved the submitted version.

## Conflict of Interest

The authors declare that the research was conducted in the absence of any commercial or financial relationships that could be construed as a potential conflict of interest.

## Footnotes

1 http://www.megasoftware.net/

2 http://www.ncbi.nlm.nih.gov

## Supplementary Material

Supplementary Figure 1 Analysis to calculate the optimal viral load for HBV genotyping **(A)** and cut-off age for detecting triple-mixed infection **(B)** in patients with HIV. SP, specificity; SE, sensitivity.

Supplementary Table 1 Sequence of primers used in nested PCR, multiplex PCR and DNA sequencing.

Supplementary Table 2 Sequence of primers used in nested PCR, multiplex PCR and DNA sequencing.
