# Supplementary figures and images for: Hepatitis B Virus (HBV) Genotype Mixtures, Viral Load, and Liver Damage in HBV Patients Co-infected With Human Immunodeficiency Virus
Source: Front Microbiol. 2021 Mar 3;12:640889. doi: 10.3389/fmicb.2021.640889 (PMC7966718; doi:10.3389/fmicb.2021.640889)

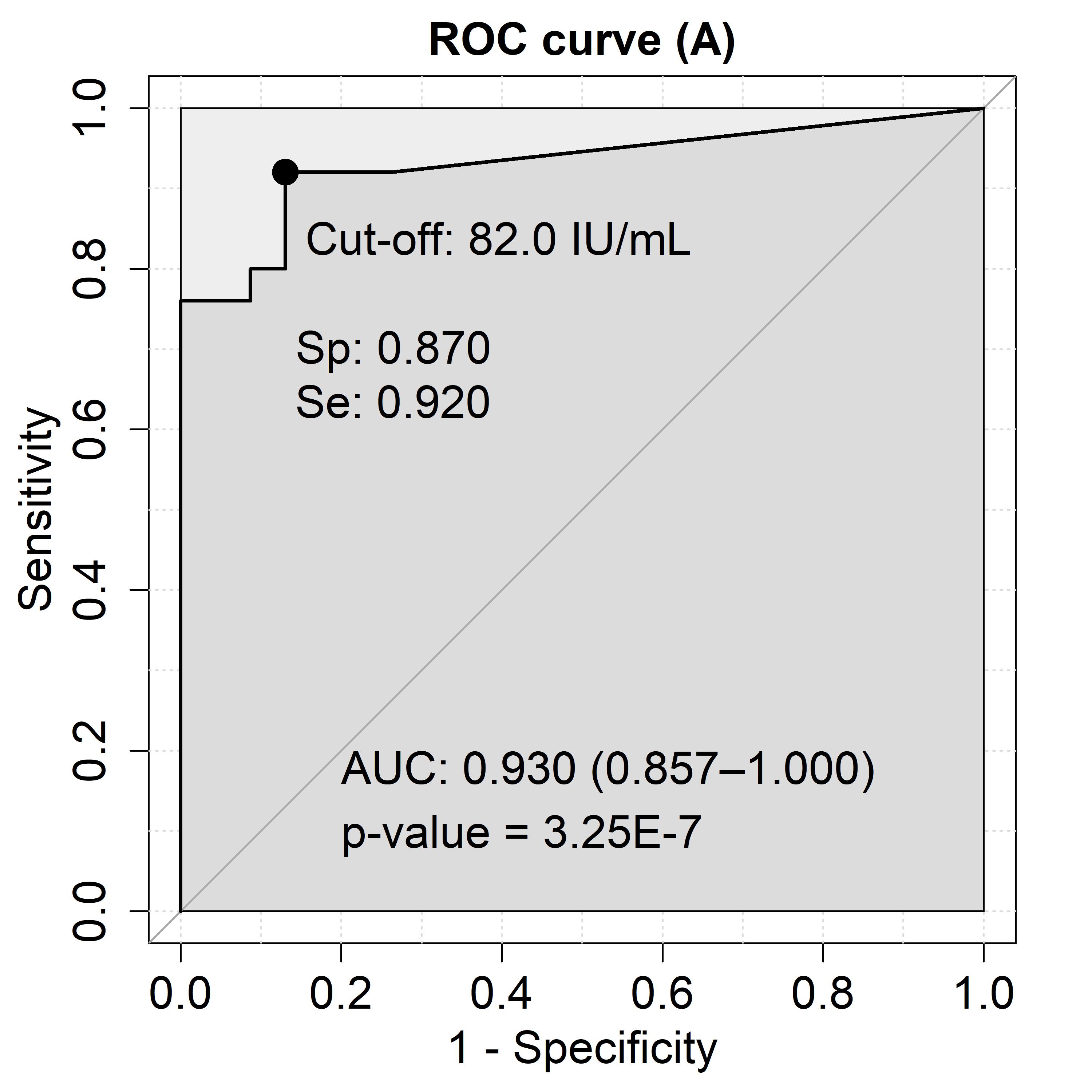

Supplement: Supplementary Figure 1 — Analysis to calculate the optimal viral load for HBV genotyping (A) and cut-off age for detecting triple-mixed infection (B) in patients with HIV. SP, specificity; SE, sensitivity. [file Image_1.JPEG]

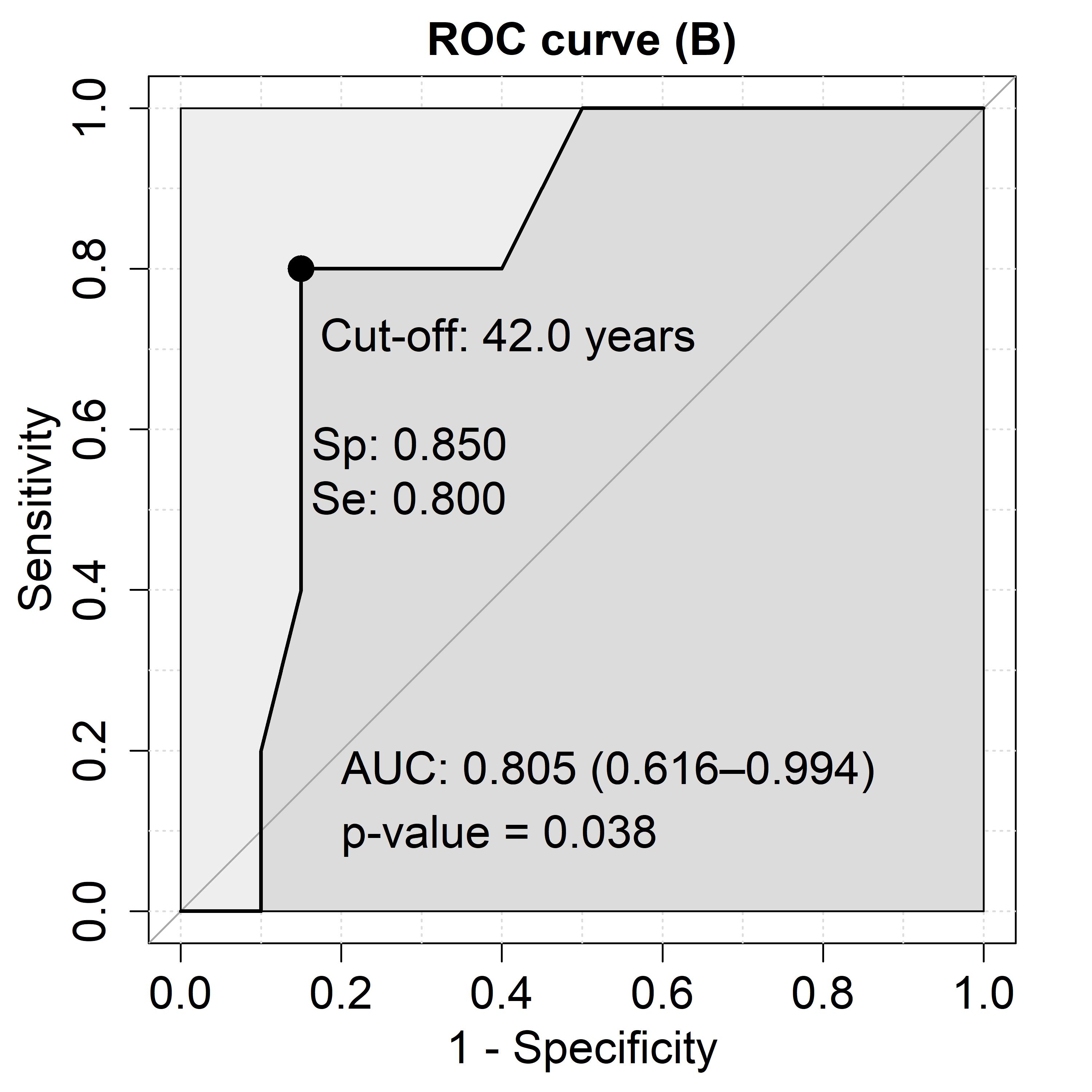

Supplement: Supplementary file 2 [file Image_2.JPEG]
