# Supplementary material for: Hepatitis B Virus (HBV) Genotype Mixtures, Viral Load, and Liver Damage in HBV Patients Co-infected With Human Immunodeficiency Virus
Source: Front Microbiol. 2021 Mar 3;12:640889. doi: 10.3389/fmicb.2021.640889 (PMC7966718; doi:10.3389/fmicb.2021.640889)
Supplement: Supplementary Table 1 — Sequence of primers used in nested PCR, multiplex PCR and DNA sequencing. [file Table_1.docx]

Supplementary Material

| **Supplementary Table 1**. Sequence of primers used in nested PCR, multiplex PCR and DNA sequencing | | | | |  |
| --- | --- | --- | --- | --- | --- |
| **Test** | **Primer** | **Sequence 5'-3'** | **Position*** | **Size (bp)** | **Gene** |
| Nested PCR | DS7-F | TCCTGCTGGTGGCTCCAGTT | 55-74 | 418 | Surface |
|  | DS8-R | CAAACGGGCAACATACCTTG | 474-455 |  |  |
|  | MS1-F | GGACCCCTGCTCGTGTTACA | 182 – 201 | 232 | Surface |
|  | MS2-R | CAGGATGAAGAGGAA(T/G)ATGA | 415–396 |  |  |
| Multiplex PCR or DNA Seq | A1-F | CGGAAACTACTGTTGTTAGACGACGGGAC | 2331-2360 | 370 | Polymerase |
|  | A2-R | AATTCCTTTGTCTAAGGGCAAATATTTAGTGTGGG | 2701-2665 |  |  |
|  | D1-F | ACAGCATGGGGCAGAATCTTTCCACCAG | 2843-2870 | 147 | Polymerase |
|  | D2-R | CCTACCTTGTTGGCGTCTGGCCAGG | 2990-2966 |  |  |
|  | G1-F | TTTGCCATATGGCCTTTTTGGCTTAGACATTG | 1912-1943 | 584 | Core |
|  | G2-R | CCAAGGAATACTAACATTGGGAAGCTGGAGATGCAG | 2498-2463 |  |  |
|  | H1-F | CTACAGCATGGGAGCACCTCTCTCMACGGC | 2844-2873 | 279 | Polymerase |
|  | H2-R | GTGGATCKGGTGGCGAGGTTGTCAGAATGC | 3123-3098 |  |  |
| bp: base pairs. **F**: Forward primer. **R**: Reverse primer. *Position based on EcoRI enzyme cleavage. | | | | |  |
