# Supplementary material for: Hepatitis B Virus (HBV) Genotype Mixtures, Viral Load, and Liver Damage in HBV Patients Co-infected With Human Immunodeficiency Virus
Source: Front Microbiol. 2021 Mar 3;12:640889. doi: 10.3389/fmicb.2021.640889 (PMC7966718; doi:10.3389/fmicb.2021.640889)
Supplement: Supplementary Table 2 — Sequence of primers used in nested PCR, multiplex PCR and DNA sequencing. [file Table_2.docx]

Supplementary Material

| **Supplementary Table 2.** **Characteristics of patients with and without HBV genotype mixtures** | | | | | |
| --- | --- | --- | --- | --- | --- |
| Variable | **Mono**  **(N=11)** | **Dual**  **(N=9)** | **Triple**  **(N=5)** | **Total**  **(N=25)** | **p-value** |
| **Gender** |  |  |  |  |  |
| Female | 0 (0.0%) | 0 (0.0%) | 1 (20.0%) | 1 (4.0%) | 0.200^1^ |
| Male | 11 (100.0%) | 9 (100.0%) | 4 (80.0%) | 24 (96.0%) |  |
| **Age (years)** |  |  |  |  |  |
| Median (Q1, Q3) | 29.0 (27.5, 36.0) | 37.0 (34.0, 41.0) | 43.0 (43.0, 47.0) | 35.0 (29.0, 43.0) | 0.035^2^ |
| **Age (class)** |  |  |  |  |  |
| < 15 | 0 (0.0%) | 0 (0.0%) | 0 (0.0%) | 0 (0.0%) | - |
| 15-20 | 0 (0.0%) | 0 (0.0%) | 0 (0.0%) | 0 (0.0%) | - |
| 21-25 | 2 (18.2%) | 0 (0.0%) | 0 (0.0%) | 2 (8.0%) | 0.670^1^ |
| 26-30 | 4 (36.4%) | 1 (11.1%) | 0 (0.0%) | 5 (20.0%) | 0.220^1^ |
| 31-35 | 2 (18.2%) | 3 (33.3%) | 1 (20.0%) | 6 (24.0%) | 0.832^1^ |
| 36-40 | 2 (18.2%) | 2 (22.2%) | 0 (0.0%) | 4 (16.0%) | 0.804^1^ |
| 41-45 | 0 (0.0%) | 1 (11.1%) | 2 (40.0%) | 3 (12.0%) | 0.080^1^ |
| 46-50 | 1 (9.1%) | 1 (11.1%) | 2 (40.0%) | 4 (16.0%) | 0.374^1^ |
| 51-55 | 0 (0.0%) | 1 (11.1%) | 0 (0.0%) | 1 (4.0%) | 0.560^1^ |
| 56-60 | 0 (0.0%) | 0 (0.0%) | 0 (0.0%) | 0 (0.0%) | - |
| 61-65 | 0 (0.0%) | 0 (0.0%) | 0 (0.0%) | 0 (0.0%) | - |
| >66 | 0 (0.0%) | 0 (0.0%) | 0 (0.0%) | 0 (0.0%) | - |
| **cART** |  |  |  |  |  |
| Without cART | 3 (27.3%) | 2 (22.2%) | 1 (20.0%) | 6 (24.0%) | 0.940^1^ |
| With cART | 8 (72.7%) | 7 (77.8%) | 4 (80.0%) | 19 (76.0%) |  |
| **HBV viral load (IU/mL)** |  |  |  |  |  |
| Median (Q1, Q3) | 23442.0 (3875.0, 154617.5) | 1479.0 (89.0, 2884.0) | 31623.0 (26915.0, 154881662.0) | 13183.0 (1479.0, 123027.0) | 0.037^2^ |
| **HIV viral load (IU/mL)** |  |  |  |  |  |
| Median (Q1, Q3) | 493.0 (37.5, 15650.0) | 40.0 (40.0, 89500.0) | 116000.0 (39.0, 328000.0) | 110.0 (39.0, 89500.0) | 0.551^2^ |
| **ALT IU/L** |  |  |  |  |  |
| Median (Q1, Q3) | 63.5 (33.0, 90.8) | 29.0 (21.0, 31.0) | 67.0 (59.0, 74.0) | 47.5 (25.2, 76.5) | 0.106^2^ |
| **AST IU/L** |  |  |  |  |  |
| Median (Q1, Q3) | 40.5 (34.0, 66.8) | 25.0 (19.0, 29.0) | 47.0 (45.0, 110.0) | 35.0 (25.8, 58.2) | 0.013^2^ |
| **GGT IU/L** |  |  |  |  |  |
| Median (Q1, Q3) | 41.0 (31.0, 49.0) | 62.0 (32.5, 70.0) | 29.0 (27.0, 275.0) | 41.0 (29.0, 73.0) | 0.940^2^ |
| **ALP IU/L** |  |  |  |  |  |
| Median (Q1, Q3) | 110.0 (98.5, 123.5) | 91.0 (69.0, 141.5) | 88.0 (70.0, 119.0) | 100.0 (80.8, 128.5) | 0.675^2^ |
| **Platelets cells/µL** |  |  |  |  |  |
| Median (Q1, Q3) | 238.0 (204.0, 290.0) | 230.0 (212.0, 269.0) | 138.0 (127.0, 168.0) | 214.0 (192.0, 279.0) | 0.048^2^ |
| **Albumin g/dL** |  |  |  |  |  |
| Median (Q1, Q3) | 3.7 (3.5, 4.1) | 3.8 (2.7, 3.9) | 3.7 (3.2, 3.8) | 3.7 (3.2, 4.0) | 0.651^2^ |
| **CD4 cells/mm3** |  |  |  |  |  |
| Median (Q1, Q3) | 262.0 (191.0, 334.5) | 273.0 (219.0, 405.0) | 189.0 (136.0, 237.0) | 251.0 (189.0, 363.0) | 0.402^2^ |
| **CD8 cells/mm3** |  |  |  |  |  |
| Median (Q1, Q3) | 838.0 (579.0, 997.0) | 895.0 (570.0, 1004.0) | 1343.0 (1188.0, 1348.0) | 957.0 (595.0, 1219.0) | 0.134^2^ |
| **APRI value** |  |  |  |  |  |
| Median (Q1, Q3) | 0.5 (0.3, 0.9) | 0.3 (0.2, 0.3) | 0.9 (0.9, 3.4) | 0.4 (0.3, 0.9) | 0.026^2^ |
| **FIB-4 value** |  |  |  |  |  |
| Median (Q1, Q3) | 0.8 (0.6, 1.3) | 0.8 (0.7, 1.1) | 2.2 (1.7, 4.6) | 1.0 (0.7, 1.5) | 0.028^2^ |
| **Liver stiffness (Kpa)** |  |  |  |  |  |
| Median (Q1, Q3) | 5.4 (5.1, 10.3) | 7.6 (6.5, 11.1) | 29.9 (19.5, 39.0) | 7.6 (5.4, 13.2) | 0.074^2^ |
| **Liver inflammation** |  |  |  |  |  |
| ALT or AST or GGT  < 40 IU/mL | 9 (100.0%) | 4 (57.1%) | 5 (100.0%) | 18 (85.7%) | 0.034^1^ |
| ALT or AST or GGT  ≥ 40 IU/mL | 0 (0.0%) | 3 (42.9%) | 0 (0.0%) | 3 (14.3%) |  |
| **APRI class** |  |  |  |  |  |
| Without significant liver fibrosis (<0.7) | 7 (70.0%) | 8 (88.9%) | 1 (20.0%) | 16 (66.7%) | 0.040^1^ |
| With significant liver fibrosis (≥ 0.7) | 3 (30.0%) | 1 (11.1%) | 4 (80.0%) | 8 (33.3%) |  |
| **FIB4 class** |  |  |  |  |  |
| Without advanced fibrosis (< 3.25) | 10 (100.0%) | 9 (100.0%) | 3 (60.0%) | 22 (91.7%) | 0.036^1^ |
| With advanced fibrosis (≥ 3.25) | 0 (0.0%) | 0 (0.0%) | 2 (40.0%) | 2 (8.3%) |  |
| **Liver stiffness class** |  |  |  |  |  |
| F1 | 6 (66.7%) | 3 (42.9%) | 0 (0.0%) | 9 (47.4%) | 0.227^1^ |
| F2 | 0 (0.0%) | 2 (28.6%) | 1 (33.3%) | 3 (15.8%) | 0.152^1^ |
| F3 | 1 (11.1%) | 0 (0.0%) | 0 (0.0%) | 1 (5.3%) | 1.000^1^ |
| F4 | 2 (22.2%) | 2 (28.6%) | 2 (66.7%) | 6 (31.6%) | 0.327^1^ |
| Frequency is expressed as number (percentage). ^1^Fisher Exact test. ^2^ Kruskal-Wallis rank sum test. **cART**, combination antiretroviral therapy; **HBV**, Hepatitis B Virus; **HIV**, Human Immunodeficiency Virus; **ALT**, Alanine Aminotransferase; **AST**, Aspartate Aminotransferase; **GGT**, Gamma-Glutamyl Transferase; **ALP**, Alkaline Phosphatase; **CD4**, lymphocyte CD4 count; **CD8**, lymphocyte CD8 count. **APRI**, AST to Platelet Radio Index; **FIB-4**, Fibrosis-4 score; **Q1**, Quartile; **Q3**, Quartile 3. | | | | | |
